# Translating research in elder care: an introduction to a study protocol series

**DOI:** 10.1186/1748-5908-4-51

**Published:** 2009-08-10

**Authors:** Carole A Estabrooks, Alison M Hutchinson, Janet E Squires, Judy Birdsell, Greta G Cummings, Lesley Degner, Debra Morgan, Peter G Norton

**Affiliations:** 1 Faculty of Nursing, University of Alberta, Edmonton, Alberta, Canada; 2 Haskayne School of Business, University of Calgary, Calgary, Alberta, Canada; 3 Faculty of Nursing, University of Manitoba, Winnipeg, Manitoba, Canada; 4 Canadian Centre for Health and Safety in Agriculture (CCHSA), University of Saskatchewan, Saskatoon, Canada; 5 Faculty of Medicine, University of Calgary, Calgary, Alberta, Canada

## Abstract

**Background:**

The knowledge translation field is undermined by two interrelated gaps – underdevelopment of the science and limited use of research in health services and health systems decision making. The importance of context in theory development and successful translation of knowledge has been identified in past research. Additionally, examination of knowledge translation in the long-term care (LTC) sector has been seriously neglected, despite the fact that aging is increasingly identified as a priority area in health and health services research.

**Aims:**

The aims of this study are: to build knowledge translation theory about the role of organizational context in influencing knowledge use in LTC settings and among regulated and unregulated caregivers, to pilot knowledge translation interventions, and to contribute to enhanced use of new knowledge in LTC.

**Design:**

This is a multi-level and longitudinal program of research comprising two main interrelated projects and a series of pilot studies. An integrated mixed method design will be used, including sequential and simultaneous phases to enable the projects to complement and inform one another. Inferences drawn from the quantitative and qualitative analyses will be merged to create meta-inferences.

**Outcomes:**

Outcomes will include contributions to (knowledge translation) theory development, progress toward resolution of major conceptual issues in the field, progress toward resolution of methodological problems in the field, and advances in the design of effective knowledge translation strategies. Importantly, a better understanding of the contextual influences on knowledge use in LTC will contribute to improving outcomes for residents and providers in LTC settings.

## Introduction

In this issue of Implementation Science we present study protocols for the Translating Research in Elder Care (TREC) program of research. We include an overview of the program (this paper), as well as protocols for the two major interrelated projects within the TREC program which were launched in 2008 and 2009 [1,2].

### Program description

The TREC research program described here is the second phase (2007 to 2012) of a long-term investigation into the determinants and processes of using research knowledge to improve care and management in healthcare organizations. The purpose of this program is to develop a robust theoretical understanding of knowledge translation in action in order to facilitate changes that result in better outcomes for recipients of healthcare. The purpose of the present five-year phase of the program is to address the impact of organizational context (*i.e*., organizational setting and environmental factors) on knowledge translation, and the subsequent impact of knowledge translation on resident health outcomes (and secondarily on provider and system outcomes) in long-term care (LTC) facilities (nursing homes) in Canada's three Prairie Provinces. In this protocol series, we primarily use the term 'knowledge translation'. While we are aware of important differences in meaning between terms and of significant terminology confusion in the field [3,4], we use the terms knowledge translation, knowledge utilization, research implementation, and research utilization synonymously in this protocol series.

### Program aims

The TREC program is guided by three objectives:

1. To contribute to the development of empirically based knowledge translation theory by examining the role of organizational context in influencing knowledge use in LTC settings, and among regulated and unregulated caregivers. This will be accomplished by:

a. Developing and implementing an organizational monitoring system to profile context in LTC facilities

b. Collecting in-depth organizational data including process changes over time

2. To pilot innovative knowledge translation interventions

3. To enhance use of new knowledge in LTC

Secondary objectives of the TREC program are:

1. To develop research capacity through the training of graduate students and postdoctoral fellows

2. To cultivate a community of decision makers in LTC in the Canadian Prairie Provinces with an interest in enhancing the use of research findings to improve resident care

3. To define ongoing objectives for the next phase of the program

The present TREC Program is multi-disciplinary, multi-level (provinces, regions, facilities, units within facilities, individuals) and longitudinal (five year), and is comprised of two main interrelated projects and a series of pilot studies. The pilot studies involve developing and assessing the feasibility of knowledge translation interventions in the areas of: strategic storytelling, supportive supervision, and leadership development. The two major projects are:

1. Project one: Building context, an organizational monitoring system in LTC.

2. Project two: Building context, a case study program in LTC.

### Project one: Building context – an organizational monitoring system in LTC

This project will monitor and explore organizational context over the five years in 36 nursing homes in the Canadian provinces of Alberta, Saskatchewan, and Manitoba. Structural facility and unit-level data will be collected through short structured interviews. In addition, unregulated (*i.e*., healthcare aides), regulated (*i.e*., nurses, physicians, allied health, educators/specialists) care providers together with managers in each facility will be asked to complete the TREC survey, a suite of instruments designed to measure organizational context and its impact on knowledge translation (three times). Data on resident outcomes will be derived from administrative data routinely collected with the Resident Assessment Instrument/Minimum Data Set – Version 2.0 (RAI-MDS 2.0).

### Project two: Building context – a case study program in LTC

This project will use an in-depth case study approach to explore the role of organizational context in promoting knowledge translation. The project will begin with comprehensive case studies in three facilities and then undertake focused case studies in six additional facilities. The facilities to be studied are all enrolled in project one. The data will be obtained through direct observation, interviews with stakeholders (care providers, provincial health leaders, managers, family members and external community representatives) and document analysis.

The purpose of this paper is to provide an overview of the TREC program at large, describing aspects of the program that are common across projects one and two, details of which are provided in the accompanying protocols [1,2].

## Background

There are two significant and interrelated gaps in the knowledge translation field. First, the science is seriously underdeveloped. Second, the use of research in health services and health systems decision making remains low, despite increased accessibility and awareness on the part of clinicians and decision makers [5-10]. These gaps have resulted in a renewed examination of the assumptions underpinning the knowledge translation paradigm. Glaser *et al*.'s encyclopaedic review of the literature on the topic [11], as well as more recent reviews [12-17], have identified major conceptual and methodological issues facing investigators in the field today. Those pertinent to the TREC program include:

1. Inadequate conceptualizations of knowledge translation, and a lack of testable knowledge translation theory (what we would term mid-range theory)

2. Over-reliance on rational actor explanatory models, and a related need to use models that focus more on organizational issues, interaction, and linkage

3. Inadequate measurement of knowledge translation, either as a dependent or independent variable

4. Lack of causal analyses

5. Over-examination of knowledge translation as product, rather than process

6. Need for inclusion of variables related to social and relational capital, and linkage mechanisms

7. Fragmentation in the knowledge translation field, and an attendant need for programmatic investigations and integration of disciplines

Findings from the first five-year phase of our program [18-22] and work by others [23-27] point to the central importance of organizational context in both theory development and successful research implementation. In the TREC program we propose to expand on this knowledge base by examining knowledge translation in the LTC sector. As in many countries, Canada has been implementing the RAI-MDS 2.0 in this sector. This implementation in LTC is sufficiently advanced to allow us to access data on resident outcomes [28-30].

### Sector definitions

Long-Term Care: In Canada we have many terms for settings providing facility-based care for the elderly [31]. In TREC, we are focusing on facility-based settings where residents live permanently with round-the-clock housekeeping, personal, and healthcare services. Whether public, voluntary, or private, we describe all of these facilities as LTC settings or nursing homes.

Workers in LTC: The care delivered to residents of nursing homes is provided largely by unregulated workers. For example, 70% of direct-care staff in Alberta nursing homes are healthcare aides (sometimes called personal or resident care aides), "an unregulated group of workers trained on the job, and students and graduates of PCA certification programs at colleges and vocational schools, which vary from 12 to 40 weeks" [[32], p. 23]. Another 13% are licensed practical nurses (LPNs) while only 17% are registered nurses (RNs).

### Why are we studying LTC?

There is a significant burden at the present time that will increase in the near future. In 2006, 13.7% of the Canadian population was over 65 years of age [33]; this proportion is expected to rise to 14.4% by 2011 and nearly 23.4% by 2031 [34]. This represents a dramatic demographic shift with consequences for all aspects of individual, community, and national life. Increasingly, we see calls for aging as a priority area in Canadian health research [35,36].

### Who lives in LTC – chronicity, frailty, vulnerability

The proportion of older Canadians who live in LTC facilities has remained stable at about 4.5% over the last two decades, and as the number of older adults in the population rises, so does the number of older adults in LTC [37,38]. Some have estimated that 43% of Canadian seniors will live three to four years in a LTC facility, and that one in five will live there more than five years [39,40]. Over one-third (37.6%) of Canadians in LTC facilities are the frail elderly over 85 years of age [41]. These residents are highly vulnerable with complex needs in all spheres and high dependency on their providers.

### Quality of care

Several reports at the international [42], national [43], and provincial levels [32,44] describe the suboptimal quality of care in LTC settings. In order to improve health-related quality of life and care of older Canadians who reside in LTC, it will be necessary to, among other things, efficiently and effectively translate research findings into better care provision, facility management, and policy making. Such translation will only occur at the provider level if those providers have both the knowledge and the capacity to use it, as well as the appropriate structural and system supports. In Canada, the training of providers in seniors' care is regarded as deficient [35]. The fact that care is largely provided by unregulated caregivers affects both the ability to apply new knowledge in practice and the quality of services offered to seniors. Better knowledge translation among all LTC providers, regulated and unregulated, in nursing homes is one important way to improve the quality of care received by seniors.

### Research considerations in relation to the LTC sector

LTC facilities have several features which make them suitable environments for studying knowledge translation. First, the facilities have sufficient but not excessive variation in critical variables, such as resident population, size, funding models, and the like across the Prairie Provinces. Second, the workforce includes both regulated and unregulated caregivers. Little attention has been paid to knowledge translation in the latter group. Third, the implementation of the RAI MDS-2.0 system is sufficiently advanced in the prairies to allow us stable outcome assessments at the resident level. Fourth, little knowledge translation work has been undertaken in these settings, and systematic efforts to improve quality of care lag behind those in acute and primary care. Balanced against this is a serious lack of resources in this sector, suggesting an urgent need for innovative interventions, designed to maximize impact and minimize resource demands. These features create a valuable natural laboratory within which to study important contextual differences and develop innovative strategies to improve knowledge translation.

### Data management

Data will be collected in a variety of forms during the course of the TREC research program. Data collected during project one will be generated from web-based surveys (regulated care providers and managers) and computer-assisted personal interviews (CAPI) for unregulated care providers. Facility- and unit-level data will be collected in structured interview format using standardized forms. Resident data will be obtained from the custodians of the routinely collected RAI-MDS 2.0. Project two data will be collected using non-participant observation, focus groups, one-to-one interviews, family diaries, facility and unit documents, and photographs of relevant artefacts and/or non-resident care activities. Details of the data types and the collection methods used in both projects one and two are contained in the respective protocols [1,2]. Protocols for the management and storage of the various forms of data have been developed.

All data will be stored on secure servers at the University of Alberta and will be managed centrally in accordance with agreed to Canadian Tri-Council standards [45]. Data quality will be ensured through standard quality control methods. Explicit procedures for checking the data for quality have been developed and are executed on a routine basis, and deviations from expected quality are investigated using defined processes. Final master and index files are designated from which all analyses will be conducted. A TREC-specific data unit has been established and is currently staffed by two data analysts, a data manager, and two trainees. Members of the data unit receive their overall direction from the principal investigator and the Data Management Committee of TREC. The TREC data manager is responsible for the secure transfer of all data from the website to the central study server at the University of Alberta. We will preserve full anonymized records of all data from the TREC research program, in accordance with CIHR's open access policy [46], indefinitely for ongoing analysis.

### Mixed method analysis

**Figure 1:**
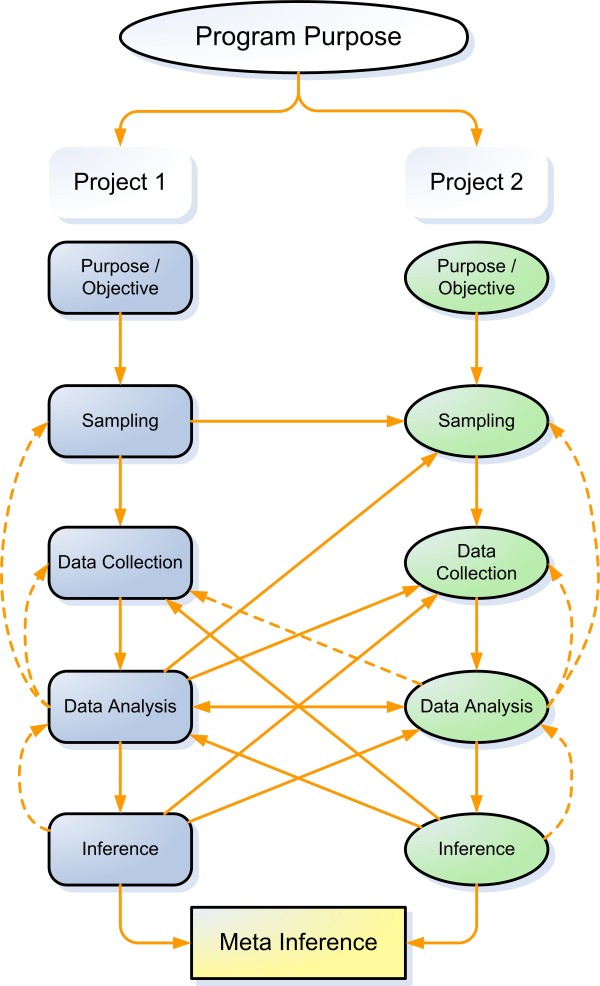
**TREC mixed method study design**. (after Tashakkori and Teddlie, 2003[47]). This Figure shows the integration of TREC projects one and two.

The TREC program draws on multiple sources of data and is based on a fully integrated mixed method design (see Figure 1) that aims to address multiple questions using quantitative (project one) and qualitative (project two) data collection and analysis methods. The broken lines in Figure 1 represent the effects of data analysis and inference development on subsequent cycles of sampling, data collection, and analysis.

The two major TREC projects are designed to complement and inform one another. Inferences will be drawn from results of the complementary qualitative and quantitative analyses and will subsequently be merged to create meta-inferences [47]. The program design includes sequential and simultaneous phases, thereby enabling the different projects and phases to inform or result in modification of parallel and sequential elements of the research program.

The use of mixed methods will enable efficient convergence on better understanding and ultimately richer theory development. Members of the project teams will shift back and forth between the quantitative and qualitative data to ensure that a robust explanation of organizational context is achieved. This will be an iterative process whereby each project can probe subsequent data collection waves of each other. For example, if the concept of organizational slack (a core dimension assessed in the TREC survey) is shown to be an important part of organizational context in project one, but has not surfaced in project two; by feeding this information to the investigators of project two, they can not only probe for it but also explore more deeply why it may be important.

The value added in this program of research comes from our ability to integrate data and analyses from multiple sources using multiple methods. The primary reason for using mixed methods is to allow us to converge on organizational context, and thus advance theory that maps organizational context and knowledge translation [47-51]. We will compare and contrast qualitative and quantitative data about the same phenomenon; emphasizing how qualitative findings link to and confirm quantitative findings, as well as how quantitative findings inform qualitative findings – thereby validating findings and enhancing generalizability. We have timed data collection to permit successive iterations so that this is possible.

Additionally, mixed methods assist in elaborating and clarifying results by capitalizing on method strengths and reducing potential method biases. This principle also applies to the examination of different levels of similar phenomena – as is the case when we undertake multi-level data collection and modeling. Combining qualitative and quantitative data and using iterative waves of data collection from multiple sources over time will provide a more complete view of organizational context.

### Ethical Approval

Ethics approvals for the two TREC main projects have been received from the appropriate university ethics boards: Universities of Alberta (#B-051007, #B-061007), Calgary (#E21379), Manitoba (#E200:010, #E200:011), Saskatchewan (BEH#08-165, 08-17), and Regina (REB#08-81).

### Return on research investment

A focus on how the results of research projects will return on the initial investment of research dollars is increasingly expected in research programs. Often this is understood as knowledge translation or integrated knowledge translation. We anticipate return will come in two main areas – the research community and the LTC sector. As such, we are engaged in both mode one (traditional scholarly activities) and mode two (partnerships with policy- and decision-makers) mechanisms of knowledge production and translation [52].

Traditional scholarly activities will include presentations at scientific conferences and publications in peer-reviewed journals, and will also occur through active participation of investigators on research and/or policy committees and groups in relevant areas.

Return on investment in the LTC sector relates to enabling timely use of relevant research findings in the LTC sector. The objectives of TREC in relation to return on investment in the LTC sector are:

1. To ensure that facilities participating in the research learn from the research undertaken in their facility in ways that are meaningful to them

2. To establish processes where all facilities within the organizations supporting TREC research (and eventually facilities in the Prairie Provinces) learn from research undertaken within TREC in ways meaningful to them

3. To contribute to creating sustainable enhanced capacity within the LTC sector to provide excellent care and support informed by research evidence

Our work has a horizon that extends beyond the five years of this study and beyond the designated study sites. This long-term endeavour will be done in collaboration with the sector; TREC can be considered one catalyst for a larger vision related to system improvements. In the short term, we will focus on the study facilities, with a view to enabling enhanced research use in facilities in the three involved provinces. Mechanisms to work toward this vision include:

1. Engagement with decision makers: Decision makers from the three Prairie Provinces are involved as partners in TREC. They participate actively in planning and strategic sessions. Strategies are also being developed in collaboration with the sector to get direct input from frontline staff.

2. Timely sharing of research results in ways that are useful to staff in the LTC sector: Working with our sector partners, feedback and results from the research program are being shared in a regular and ongoing manner and in mutually agreed ways (*e.g*., in-person, in brief written form, using posters) with both site administrators and frontline staff.

3. Collaboration with organizations with a mandate or interest in enhancing research use capacity: Participating in one research program will not ensure ongoing capacity enhancement. Discussions are ongoing with organizations that are interested in collaborating to build sustainable capacity to use research findings in the LTC sector (*e.g*., Health Quality Councils of Alberta and Saskatchewan, Health Canada).

4. Strategic dissemination: This will include dissemination of 'plain language' results and other information relevant to policy makers and organizations that influence care of the elderly. Strategic dissemination will be planned by the research team and sector partners, jointly.

### Capacity building

We have an opportunity to build research capacity in both the knowledge translation and LTC fields by providing an enriched training environment. Several trainees at both the doctoral and post-doctoral levels are currently engaged in the research program, and other trainees will be recruited throughout the duration of the program. When feasible, they will be actively involved in aspects of the program beyond their own work, such as project administration and interaction with decision makers.

## Conclusion

The products resulting from the various projects described here will contribute to: integration and theory development across disciplines; identifying and resolving major conceptual problems in the field, similar to Van de Ven's work [53] in innovation; resolving major methodological problems in the field; and advances in how to design effective knowledge translation strategies for the burgeoning group of unregulated individuals caring for seniors in LTC. The TREC program is about advancing understanding of how organizational context affects knowledge translation. We have chosen to do this in LTC because it presents a unique laboratory for study in knowledge translation. Nursing homes are also places in which some of Canada's most vulnerable citizens live and in which health system improvements are urgently needed.

## Competing interests

The authors declare that they have no competing interests.

## Authors' contributions

CAE is the principal investigator for the TREC research program. She conceived the program and its design, secured its funding, is providing the leadership and coordination for the program, and provided substantial commentary to the final submitted manuscript. AMH and JES are trainees within the TREC research program and made significant contributions to drafting the manuscript. DM, GGC, LD, JMB and PGN participated in designing the study, securing grant funding, and provided critical commentary to the final submitted manuscript. CAE and PGN co-lead project one and LD is the lead investigator for TREC project two. LD, DM, GGC are provincial site leads for Manitoba, Saskatchewan, and Alberta respectively. All authors read and approved the final submitted manuscript.
